# SmarTEG: An Autonomous Wireless Sensor Node for High Accuracy Accelerometer-Based Monitoring

**DOI:** 10.3390/s19122747

**Published:** 2019-06-19

**Authors:** Michele Magno, Lukas Sigrist, Andres Gomez, Lukas Cavigelli, Antonio Libri, Emanuel Popovici, Luca Benini

**Affiliations:** 1 Integrated Systems Laboratory, ETH Zurich, 8092 Zurich, Switzerland; lukas.sigrist@tik.ee.ethz.ch (L.S.); gomeza@iis.ee.ethz.ch (A.G.); cavigelli@iis.ee.ethz.ch (L.C.); a.libri@iis.ee.ethz.ch (A.L.); benini@iis.ee.ethz.ch (L.B.); 2 DEI, University of Bologna, 40126 Bologna, Italy; 3 School of Engineering, University College Cork, T12 K8AF Cork, Ireland; e.popovici@ucc.ie

**Keywords:** WSN, IWSN, data processing, wear detection, low power wireless solution, energy harvesting, energy neutral systems

## Abstract

We report on a self-sustainable, wireless accelerometer-based system for wear detection in a band saw blade. Due to the combination of low power hardware design, thermal energy harvesting with a small thermoelectric generator (TEG), an ultra-low power wake-up radio, power management and the low complexity algorithm implemented, our solution works perpetually while also achieving high accuracy. The onboard algorithm processes sensor data, extracts features, performs the classification needed for the blade’s wear detection, and sends the report wirelessly. Experimental results in a real-world deployment scenario demonstrate that its accuracy is comparable to state-of-the-art algorithms executed on a PC and show the energy-neutrality of the solution using a small thermoelectric generator to harvest energy. The impact of various low-power techniques implemented on the node is analyzed, highlighting the benefits of onboard processing, the nano-power wake-up radio, and the combination of harvesting and low power design. Finally, accurate in-field energy intake measurements, coupled with simulations, demonstrate that the proposed approach is energy autonomous and can work perpetually.

## 1. Introduction

Over two-thirds of the total electrical energy generated in the United States is estimated to be consumed by motor-driven systems [1]. Increasing the energy efficiency of these systems is crucial, and good maintenance can be the key to reaching this objective. As reported in [2], industries could save up to 18% of the energy consumed if motors were ran under healthy conditions. For example, in a machining farm, due to attrition, thermal fracturing, material deformation, and abrasion, the cutting tool gradually wears out, loses its sharpness, and becomes blunt. This affects the machining process, the health of the machine tool and significantly increases the power consumption. Finally, it can also have health and safety implications. All these factors have a direct impact on productivity and the cost of the final product. 

Traditionally, industrial automation systems are realized through wired communication, and real-time sensor data acquisition and control is common. Collaborative, low power and low-cost wireless sensor networks (WSNs) can reduce infrastructure expenses, facilitate installation and cut maintenance cost. WSNs can render smart monitoring economically interesting for new application scenarios, thus enhancing today’s automation systems that enable energy savings and reduce CO_2_ emissions by optimizing the management of industrial systems. In this case, WSNs are commonly referred to as Industrial Wireless Sensor Networks (IWSNs) [3]. The sensors of an IWSN can be deployed in oil pumps, in the heart of moving engines, and vibration sensors on packing crates and unsafe, inaccessible or hazardous environments which cannot be accessed with normal wired systems.

WSNs are more effective than periodic manual check-ups since they offer centralized monitoring and automatic failure recognition with a low-cost solution [4]. Accelerometers are currently among the most widely studied MEMS sensors in a wide range of applications: medical, sports and recreation, entertainment, industrial, etc [2]. This is due to their accuracy in the detection of vibrations and human body or machine movements, their small size, and reasonable power consumption [4,5,6]. Recent works have described the use of accelerometers in specific applications of wireless sensor networks for motor analysis and machine tool performance. IWSNs with accelerometers and other sensors enable condition monitoring systems for small electric motors [5], a replacement for traditional motor vibration monitoring sensors as well as alarm systems for fault detection [7]. 

IWSNs are usually battery operated, making low power consumption a critical requirement for long-lasting operation. It is commonly accepted that wireless communication represents the largest share of the power consumption in such devices. Smart WSNs process the data on-board and detect a fault or pattern locally, sending an alert only when necessary. Energy harvesting technologies provide a possible solution to alleviate WSN battery lifetime issues further. Ambient energy is harvested from vibration, temperature difference and light [8,9,10,11,12,13] to power the WSN. In the case of Radio Frequency energy harvesting, which is a new promising approach, the ambient energy can be provided by commercial RF broadcasting stations, such as TV, GSM, Wi-Fi, or radar [14,15,16,17]. In general, the power management circuit charges a supercapacitor, which is then discharged to utilize the energy. The utilization of perpetual environmental energy leads to a long lifetime for WSN systems. Multiple sources of energy harvesting from more than one type have been proposed in order to maximize power generation [13,18,19].

In this paper, we present the design and implementation of a self-sustainable wireless sensor node with an accelerometer-based algorithm for wear detection of a band saw. The same principle can be applied to other electrical machines for an improved monitoring solution. The sensor node allows monitoring machinery in industrial environments where a wired connection for power or data to external sensors devices cannot always be provided [20]. Thus, in these scenarios both self-sustainability and wireless communication are crucial and often mandatory features. In this work, we use a ZigBee PRO radio, which is suited for industrial application, and improved using a nano-power wake-up radio receiver to reduce power consumption by switching off the Zigbee transceiver while maintaining the wireless capability and reactivity. The node has a 100% accuracy using an on-board tunable algorithm. To ensure perpetual energy supply, a thermoelectric harvesting subsystem is used and characterized with in-field experimental results including all the conversion stages. The main contribution of the paper is on the ultra-low power hardware and software co-design of a wireless self-sustainable sensor node. The designed node uses energy harvesting, low power hardware, wake up radio technology and an optimized, high accuracy detection algorithm. Experimental validation, using simulation and in-field experimental results demonstrate the proposed solution’s performance in terms of accuracy and energy autonomy. We used a real case of blade wear detection to have an in-field test bed to evaluate the proposed solution.

The remainder of this paper is organized as follows: in Section 2 related works are presented, while our system architecture is described in Section 3. Section 4 gives details on the detection algorithm, and Section 5 specifies the energy model for an autonomous energy system. In Section 6 we present the experimental setup and measured results in terms of accuracy, power consumption and generated power generated using the TEGs. We then combine these results to verify long-term energy neutrality by simulation in Section 7. Section 8 concludes the paper.

## 2. Related Work

In recent years, research on IWSNs has been prolific, with a variety of systems and techniques to build low cost, wireless systems to monitor tools and machinery being described. Several IWSN devices have been presented and implemented by academic researchers or by commercial organizations [20,21,22]. Many previous consider different application scenarios of IWSNs. However, most of them only use nodes for data acquisition and transmission to a remote central computer, which is in charge of feature extraction and fault diagnosis functions. Our work presents an alternative approach to raw data transmission using on-node data processing, feature extraction, and fault diagnosis. This approach can reduce the quantity of transmitted data considerably, save network and node energy, and thus extend the lifetime of the network and of the node. To date, this approach is a relatively unexplored area for industrial wireless sensor networks, and only a few research papers can be found related to sensor (smart sensor) processing [23,24,25,26]. 

Many papers, including [20,25], consider the problem of rotating machinery faults, particularly the ones that can affect electric motors, mainly because this is a common occurrence in many industrial plants. Several systems monitoring the wear state of cutting tools are presented in the literature, and various methods have been developed to identify tool wear states. Very low-cost current sensors to develop a tool wear monitoring system have been presented in [27,28]. All these works highlight that the current consumption of the cutting tools depends on the wear of the blade, with direct implications on the cost of the final product. In [23], an accelerometer, a microphone, and an acoustic sensor are used together to perform fault detection with wireless sensor networks. The authors present an interesting approach using data fusion and transferring the data only to a remote computer via a radio link after processing it. The work is very similar to the presented approach. Unfortunately, the authors in [23] did not disclose any information regarding the power consumption and implemented an algorithm to compare to the proposed system, but it is likely in the order of watts as it requires a PC, which is more than one order of magnitude higher than our approach.

Regarding power consumption, energy efficiency, and energy harvesting, research in the WSN domain has been intense in recent years with a variety of methods and techniques to extend the lifetime of networked nodes [8,9,10,11,12,13]. These approaches show the importance of the synergy of energy harvesting and power management to achieve self-sustainability in real-world applications [28,29,30,31]. Recently works on energy harvesting from TEG have been demonstrated to be able to effective also for miniaturized wearable devices [32] On the power management side, most efforts attempt to fundamentally reduce the RF energy requirements of the devices via a number of novel hardware solutions such as wake-up radios (WURs), software (i.e., media access control and routing algorithms) and duty cycle optimization approaches [17,33,34,35]. In [29,35], energy efficiency is achieved by reducing the active periods of a node through changing the duty cycle, and optimally waking the system from ‘deep sleep’ modes. It has been highlighted that extending the lifetime of networks is a strategic enabler, if not a primary requirement, for a significant number of applications, including but not limited to, the industrial field. With respect to these papers, this work presents the design, implementation of a vertical solution and its in-field characterization. 

The proposed approach combines low complexity algorithms, low power electronics, power management with wake-up radios and energy harvesting to achieve highly accurate fault detection ready to work perpetually. 

## 3. System Architecture

**Figure 1:**
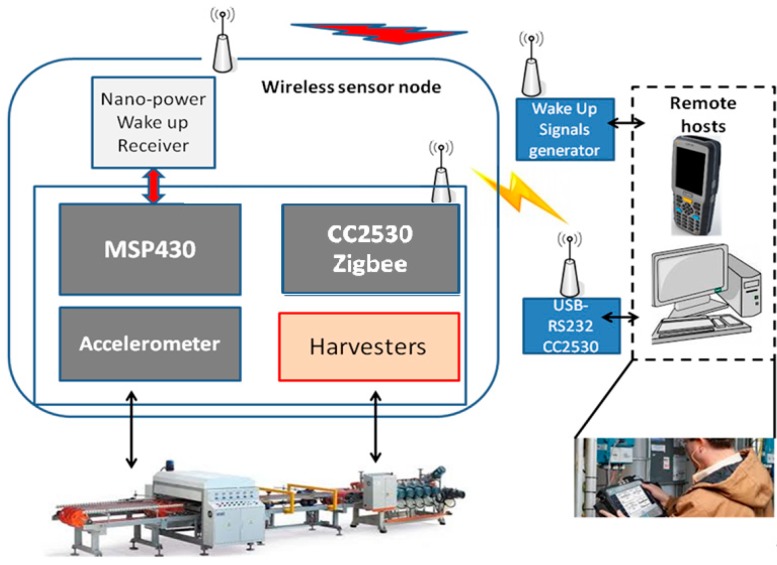
Block diagram of the sensor node and its environment [20].

As mentioned in the introductory section, various industrial processes require routine maintenance to achieve the best manufacturing performance in terms of money spent both for consumed power and for replacement parts. The application scenario of this work is monitoring machinery using an accelerometer-based algorithm implemented in a low-cost energy neutral wireless sensor node. Figure 1 shows the basic idea of this approach where the wireless sensor node is attached to the machinery to measure both energy and information about the vibrations. A three-axis accelerometer, the CMA3000-D01 from Murata (Kyoto, Japan), has been employed, especially for the low power consumption of few μW and the small size. The accelerometer is interfaced with a microcontroller through a serial interface (SPI) and is sampled at 340 Hz to obtain data for the detection algorithm. A low power microcontroller (a MSP430 from Texas Instruments, Dallas, TX, USA) processes the vibration data directly onboard. The status of the monitored machinery is analyzed, classified and transmitted to a remote host when a critical wear situation is detected. In addition, the status can be requested by the remote host. To avoid the waste of precious energy for the idle-mode listening of the onboard radio, a nano-power wake-up radio is used for asynchronous communication [8]. Figure 1 also shows the building blocks of the developed wireless sensor node. It is built around the CC2530 system-on-chip and MSP430 microcontroller from Texas Instruments and a 3-axis accelerometer connected with a serial port (SPI) to the MSP430 microcontroller. The MSP430 has been chosen for the low power consumption, which is around 310 μW when supplied at 3 V and clocked at 1 MHz, and its capabilities are sufficient for managing the bandwidth of the sensor (340 Hz sampling) as well as performing the classification algorithm and the network activities in real time. On the other hand, the CC2530 is in charge of communication with the ZigBee protocol driven by the MSP430. This ZigBee chip has been selected as it provides the Z-STACK from TI using an open-source API implemented for the MSP430. Thanks to this stack the MSP430 can easily be programmed to control the data transmission using the ZigbeePRO radio through the CC2530.

### 3.1. Energy Harvesting

Given the goal of the paper to implement an embedded, energy neutral, wireless monitoring system, we were mostly interested in the possibility to provide energy to the system during data processing. An energy harvester with thermoelectric generators (TEG) was selected for this goal. TEGs are devices that convert heat into electrical energy through the Seebeck effect. To work as a generator, one side of the TEG must have a temperature greater than the other side. As industrial machinery usually provides a continuously warm surface while they are working, TEGs are good candidates for energy harvesting. Indeed, TEGs are preferred to solar panels and vibration harvesters because machinery can work in the dark and because vibration energy is limited at low frequencies (below 20 Hz) and strongly depends on the harvester’s resonance frequency.

The energy harvesting circuit is based on the LTC3107 power management chip from Linear Technology (Milpitas, CA, USA) which harvests energy with a very low input voltage down to 20 mV. This is an important feature, as the transducer’s voltage is often only a few tens of mV. The LTC3107 can charge either a battery or a supercapacitor, and it provides an integrated high efficicent regulator to supply the node directly with 3 V. For our experimental results, we used a supercapacitor to demonstrate that self-sustainability can also be achieved with very small energy storage. Alternatively, the system could be equipped with a bigger supercapacitor or Li-Ion batteries according to the application’s requirements. Thanks to this chip it is possible to achieve a high conversion efficiency and high flexibility covering a wide range of TEGs with only a few square millimeters of space. A 10 × 8mm QC-32-0.6-1.2 TEG module with a 14 × 14 mm heat sink has been used to harvest power from the heat emitted by the machinery. The module can provide up to 40 mW with this small sink (measured at maximum power point) at ΔT = 50 °C. However, this power is achieved only with a very high delta of temperature on the TEG, which is not easily achievable in real applications. Moreover, the average harvested power is always lower than the peak value, and it is necessary to convert this power from a very low voltage (around few hundred of mV) to a higher voltage (3–5 V) needed to supply the node and charge the battery. As we will present in the experimental results section, this conversion has significant power losses (around 70–90%) which reduce the available power. For these reasons, the average power of the selected TEG in real operating conditions is in the order of 1–2 mW which matches the average power needed from the developed microcontroller that is in the same order of magnitude. 

### 3.2. Wake-Up Radio

The wake-up radio is an always-on receiver which detects wake-up messages sent from a remote host. There are many approaches to wake-up radios with different trade-offs in terms of power, range, features (addressing or not addressing) [33]. The wake-up radio significantly reduces the main radio and sensor node activities and wakes up the system only when needed. This approach helps the system be self-sustainable by reducing overall power consumption. In this work, we integrate the wake-up radio developed in [34]. It was tuned to generate an interrupt only with a wake-up message of 50 μs with on-off keying sequence of zeros (‘0’) and ones (‘1’). As our main goal is to minimize the power consumption, and since the application scenario does not need an addressing feature, the ultra-low power radio wake-up was developed with a low power comparator, which is the unique active component of the subsystem and generates the interrupt to wake up the whole system [34]. This ensures the lowest power on, which is ultimately determined by the always-on comparator. The listening power consumption measured is 600 nW @ 3 V using an AS1976 comparator [34].

## 4. Wear Detection Algorithm

As mentioned before, the sensor node must be mounted on the band saw selected for the application scenario. The research on machine fault detection is mainly focused on the use of the Fast Fourier Transformation (FFT) to identify typical patterns that can be exhibited. However, a main goal of the proposed approach is on-board processing on a low power microcontroller, such that an algorithm based on the FFT is unfeasible due to the computational effort. The Mean Absolute Value (MAV) of the vibration in the time domain is used due to its low power consumption and low complexity. On the other hand, the FFT gives a more accurate analysis when the frequency of the vibrations is higher or frequently changes during the time. The MAV can be evaluated as follows:

(1) $$MAV_{x}=\;\frac{1}{N}\;\overset{N}{\underset{i=1\;}{\sum }}\left|x\left[i\right]\right|,$$

where x[i] is the discrete value at tithe me *i* of the accelerometer data sampled in the continuous time *t*, and N is the number of samples. In relation to the sign changing of absolute value, as x[i] is a binary number on the microcontroller the two’s complement representation is adopted, and to change the sign of a number stored in memory two simple steps are required: (i) take sample x[i], (ii) calculate $y=\bar{x}\left[i\right]$, (iii) the absolute value is obtained by y=y+1, where y is a local variable and $\bar{x}\left[i\right]$ is the one’s complement of x[i]. Concerning the division of the x samples in (1) we use N=a, where a is a power of 2 (i.e., $a=2^{9}$ or $2^{8}$ or $2^{7}$). Hence, the division can be evaluated as a simple bit shift, and we can accomplish the computation with the limited resources of the microcontroller. To reduce the power consumption of data processing, the idea is to calculate the average considering only small blocks of data. *N* is chosen not higher than $2^{9}=512$ samples. The higher the *N* sample number chosen is, the longer the time to generate a MAV will be, as more data samples are required. Table 1 shows the relationship between samples and time interval and response time. 

Therefore the calculation of the MAV is performed in sequence, considering small time intervals: 1.5 s, or less. One axis of the accelerometer is enough to give an accurate classification of the wear detection. This makes the process three times faster and cheaper in terms of power consumption.

Selecting the axis to be used is done during a calibration process at the moment the node is installed on the machinery. Thus, we use only the x-axis data to classify the status of the blade. Since the MAV value can be unstable with only a single MAV as we will see in Section 6, to increase the accuracy of the algorithm the microcontroller saves the last four values of the previous MAV and evaluates the average before the classification step. The algorithm flow steps implemented are:

(1) Calculate MAV value following (1)
(2) Repeat Step 1 four times to get an average MAV
(3) Compare the MAV values with the thresholds
(4) Threshold-based classification of the wear
(5) Save the status for host requests
(6) If excessive wear is detected, send an alarm to host using the main radio

**Figure 2:**
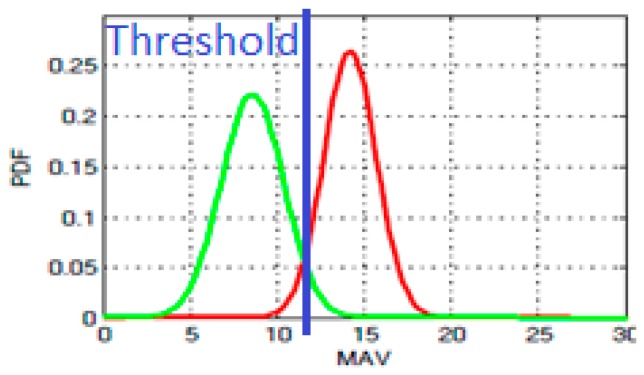
The probability density functions of the averages along one axis when cutting the brass. The green curve is the fitted Gaussian PDF for the “new blade” dataset and the red curve for the “old blade” dataset.

The threshold value is also an important parameter, as it can significantly affect the algorithm’s accuracy. To choose this value, a training phase is needed. The training phase is done off-line on the PC using an acquired dataset, i.e., it is not evaluated during the classification on the microcontroller. Figure 2 shows two Gaussian probability density functions (PDFs) fitted to the measured data along one axis and the corresponding selection of the threshold (‘new blade’ in green, ‘used blade’ in red). It can be noticed the maximum of the green curve is reached at the MAV value of 8 for the new blade and 14 for the old blade, such that the threshold for brass could be chosen optimally at 11 to distinguish between old and new blade. Other thresholds could be used to detect medium use blades, as we will show in Section 6. This process is repeated for all the materials to find the optimal thresholds. After the training period, the classification thresholds can be fixed in the run-time algorithm performed on the microcontroller. The run-time classification on the node is done with a simple decision tree with 1 level (the MAV value) and 3 leaves/classes (new/medium/old) according to 2 thresholds decided in the training phase. 

## 5. Energy Model

The energy model for the proposed application is presented below. Due to the ultra-low power design of the node, the deep-sleep status saves substantial energy when communication and sensing are not needed. Over time, the node’s overall consumed energy *E_u_* is defined as follows:

(2) $$E_{u}=\left(P_{on}\cdot T_{on}\right)+\;P_{sleep}\cdot T_{sleep},$$

where $P_{on}$ and $P_{sleep}$ are the node’s power consumption when in active mode and in sleep mode respectively, while $T_{on}$ and $T_{sleep}$ are the active and sleeping periods. Assuming periodical polling, we can define a period $T=T_{on}+T_{sleep}$. As we mentioned, the design of the node allows $P_{sleep}\ll P_{on}$, such that reducing the active time extends the lifetime of the node. However, while $T_{sleep}$ depends on the application and the user settings at runtime, the$\;T_{on}$ depends on the execution time of the application, or more precisely on the single tasks executed:

(3) $$T_{on}=\left(T_{wup}+T_{sens}+T_{proc}+T_{tx}\right),$$

where $T_{wup}$ is the time needed to change the status from sleep to active, $T_{sens}$ is the time to collect data from the sensor, $T_{proc}$ is the time needed for the processing, and $T_{tx}$ is the time for the communication. The nano-watt wake-up radio allows to switch off the main radio and microcontroller, and still provides a reactive wake-up. The wake-up radio can significantly decrease the $P_{sleep}$, reducing the overall$\;E_{u}$. When the node is equipped with the energy harvester, the intake energy in period T is:

(4) $$E_{h}=P_{TEG}\cdot T,$$

where$\;P_{TEG}$ is the average power collected from the TEG harvester over the time T. The energy balance at time T is given by:

(5) $$E_{tot}=E_{h}-E_{u}.$$

In an autonomous system, when a battery or storage is present, the system can store or use already stored energy. When$\;E_{tot}$ is positive (then$\;E_{h}>E_{u}$), the energy stored will increase while the energy storage will decrease when$\;E_{tot}$ is negative (then$\;E_{h}<E_{u}$). Conservatively assuming an ideal energy neutral system where$\;E_{u}=E_{h}$, the node is designed to have$\;P_{on}$ and$\;P_{sleep}$ as low as possible. To guarantee the cold start of the device and energy supply stability, an energy buffer can be used. In systems with energy storage, it is possible to evaluate the energy availability over time according to:

(6) $$E_{store}\left(t+1\right)=E_{store}\left(t\right)+E_{h}-E_{u}.$$

Moreover, as the energy buffer is limited,$\;E_{store}$ cannot exceed a maximum value$\;E_{max}$, which is the capacity of the storage. On the other hand,$\;E_{store}$ has to guarantee the cold start, where $\;E_{store}$ from zero needs to be greater than or equal to minimal value of storage $E_{min}$ to supply the system. For this reason, the following equation has to be satisfied for any time t:

(7) $$E_{min}\leq E_{store}\left(t\right)\leq E_{max}.$$

## 6. Experimental Results

In this section, an experimental evaluation of the system is presented. As a first step, we present the setup of the testbed used for the evaluation. We then compare the accuracy of our proposed, optimized algorithm to the FFT-based approaches. Afterward, the power consumption of the proposed device exploiting the accelerometer sensor is presented for various configurations. These measurements are a crucial parameter to understand energy consumption in the application scenario and to select the energy harvesters to achieve self-sustainability. Finally, the TEG module is characterized by different temperature gradients and the measured energy harvested. A testbed with the TEG supplying the node during the activities is evaluated to demonstrate the self-sustainability of the proposed solution.

### 6.1. Experimental Setup

**Figure 3:**
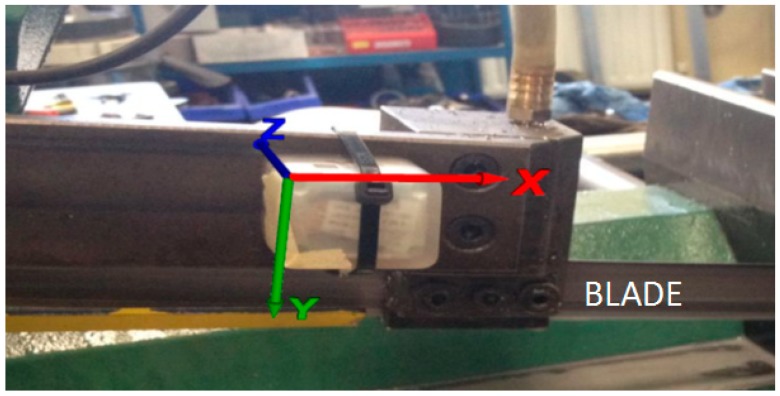
Sensor node mounted on the bandsaw’s arm and its orientation.

To evaluate the performance of the proposed system in a real application scenario, the wireless sensor node has been developed and installed directly on a band saw Figure 1 and Figure 3. The prototype developed has a size of 5 cm × 3 cm and the possibility to host a single TEG module which can be attached externally. Thus, the TEG can be placed in the warmer place to extract more energy and the node in the best place for sensing the vibration. The TEG module can then be connected to the node with two wires (GND, and V+). The WUR antenna used is an 8 cm × 1 cm, 868 MHz flexible and with only 0.10 mm height from Molex (Lisle, IL, USA) that can be mounted with the adhesive, directly on the node package. The band saw is a double speed control SN270 (Pedrazzoli, Passano, Italy). The Pedrazzoli band saw used in the laboratory was fitted with blades made by Lenox (East Longmeadow, MA, USA. The node was supplied with 3 V and with the microcontroller algorithm running at 1 MHz. To evaluate the node power consumption, we used a shunt resistor between the power supply and the node to trace all the node modes (and modes’ times) and the relative current consumption. A 100 Ω shunt resistor has been used to measure the low current modes (sleep/processing) accurately, and a 1 Ω resistor for the high current modes (wireless communication). As the voltage supply has been fixed at 3 V, it was possible to evaluate both power and energy consumption (with the modes’ times). 

As mentioned in Section 3, a TEG power generator has been used due to its small size and the possibility to guarantee the power needed from the node in the temperature range of the application scenario. The TEG module has been tested in a thermal chamber with settable temperature and a hot plate to evaluate accurately the power generated for different temperature gradients. Two small thermocouple probes have been inserted at contact with the hot surface and cold surface to measure the temperatures and evaluate the gradient. To also evaluate the real power harvested including the losses by the conversion circuit and the DC-DC converter, the same chamber has been used and the energy harvested in the storage capacitor has been measured. Finally, the node has been attached to the $V_{out}$ of the LTC3107 when the chamber had a temperature of 18 °C and the hot plate of 38 °C to evaluate the effective self-sustainability of the energy harvesting. The temperature values have been measured on the Pedrazzoli band saw used with a lab room temperature measured during the characterization was almost constant, in the range of 17–19 °C. 

### 6.2. Wear Detection Algorithm

**Figure 4:**
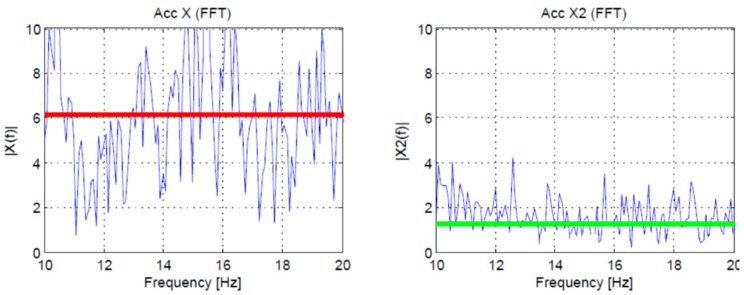
Accelerometer data in the frequency domain and mean absolute value (red and green horizontal line) evaluated in the frequency domain in the range 10–20 Hz during 10 s of brass cutting. On the left side, there are the plots for an old blade. On the right side the plots for a new blade.

We present a complete set of experimental results to evaluate the accuracy of the proposed algorithm. A comparison with FFT for several materials is presented, and all tests were performed with three different blades. One blade was new, one used for the average blade lifetime, and one was old. One hundred and twenty (120) datapoint acquisitions were made, 40 for each of the different blades on different days to increase the wear of the blade. The plots in Figure 4 highlight the significant discrepancies resulting from using a new (right side) versus an old blade (left side). The red line on the plots shows the mean absolute value calculated in the range of 10 to 20 Hz in the frequency domain, computing FFT on the accelerometer data collected from the node. 

**Figure 5:**
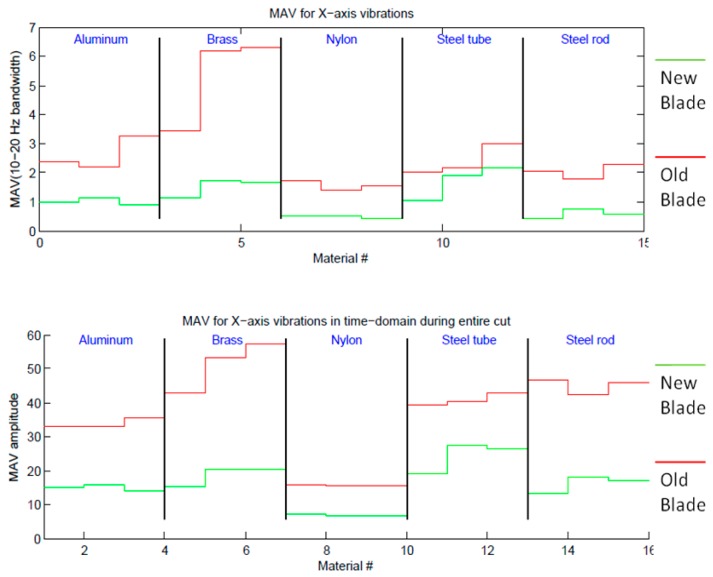
Comparison of MAV algorithm with FFT (first plot) and MAV in time domain (second plot) using five test materials in three different datapoint acquisitions for each material.

Figure 5 shows the analysis in the frequency domain where the mean average value is evaluated after performing the FFT (top plot), and in time domain plot according to (1) (bottom plot) where the MAV is evaluated on the accelerometer amplitude in the time. These data have been collected directly by the developed wireless node at a 340 Hz sample rate and then used to evaluate the accuracy of the algorithm.

Both MAV evaluations were done using only the x-axis data from the accelerometer (frequency for the first plot, amplitude in time for the second). The materials used during the dataset acquisition were aluminum, brass, nylon, steel tube and steel rod. The cut for each material was done three times, using an old blade and a new blade to evaluate the vibration in two extreme conditions. The first plot shows the MAV value in the frequency domain obtained using the data from the accelerometer, acquired at 340 Hz for 10 seconds, evaluating the mean absolute value of the FFT in the range 10–20 Hz. Both plots show that there is a large difference between vibrations produced with a new blade and an old blade. Moreover, the plots show how the frequency approach is very similar to the time domain approach, although for the limited resources of the microcontroller the time domain approach can be performed faster and with lower power consumption. On the x-axis of the plots, there are the fifteen samples evaluated (three per material). 

Table 2 shows the accuracy of the MAV with four consecutive evaluations is always 100% as is the case of the MAV in the frequency domain evaluated performing an FFT on the data from the accelerometer. It is important to notice how the low power version of the algorithm, with only a single MAV evaluation, is always 100% accurate detecting an old or new blade, but it is missing a few intermediate states reducing the accuracy down to 77% in the worst case. Thus, the single MAV evaluation, on the one hand, decreases the time and energy requirement, on the other hand, the accuracy and performance are lower. However, it is still a high accuracy alternative for low power evaluation, and this can be useful when the energy availability is low. 

To evaluate the proposed algorithm (Section 4) in terms of accuracy in comparison to the frequency domain, the MAV evaluation according to (1) with N=512 samples was used with the thresholds shown in Table 3. The results of both algorithms were compared to the MAV in the frequency domain and evaluated in terms of accuracy on a PC using MATLAB. Finally, as an example, Table 3 shows the thresholds used for the two versions of the algorithm in the case of the Nylon that was the most critical material to correctly classify as Table 2 shows. The thresholds for each material have been selected using a training dataset of 100 samples for different materials.

### 6.3. Power Consumption Measurements

Table 4 shows the sensor nodes’ current consumption in different modes measured with the shunt resistor and an InfiniVision MSOX2024A oscilloscope (Agilent Technologies, Santa Clara, CA, USA). Measurement of the wireless sensor’s power (at 3V) and energy consumption was performed assuming the node can be in one of the four configurations shown in the table. When the main radio CC2530 is not needed this is set in Low Power Mode 3 (LPM3) to save energy as the power consumption is only 1 μW. In this status, the node receives messages through the nano-watt WUR board that is connected via GPIO to give an interrupt to the MSP430 microcontroller. Since the WUR power consumption is negligible (600 nW) with respect to the power of the main radio, it is a useful technique to achieve an energy neutral node. The table shows that the radio’s power consumption is over 150 times more than the onboard data processing, so it is essential to keep the radio off and activate it only when needed. This also confirms the benefits of processing the data on board as the radio can be used only to transmit the results of the classification as opposed to all the data that was acquired.

Table 4 shows that the time taken for the algorithm to acquire 512 samples and repeat this MAV four times to have the best accuracy performance is 6 seconds. After this, it transitions to the processing state, which takes 7 ms and is thus negligible. If it would take more time, it should be considered parallelizing this processing step with the data acquisition. The time to send the information byte of the blade’s wear via radio is around 382 ms. For this reason, the energy consumption is 2.79 mJ for the higher accuracy algorithm, 0.696 mJ for the lower accuracy fast response algorithm and 39 mJ for the transmission. As the transmission contains only the information of the blade status (new-old-medium) and not the raw data of the vibration, the transmission energy is independent of the algorithm used with four MAV or a single MAV. In this way, the energy for transmission, as we saw it is dominant, is minimized. 

**Figure 6:**
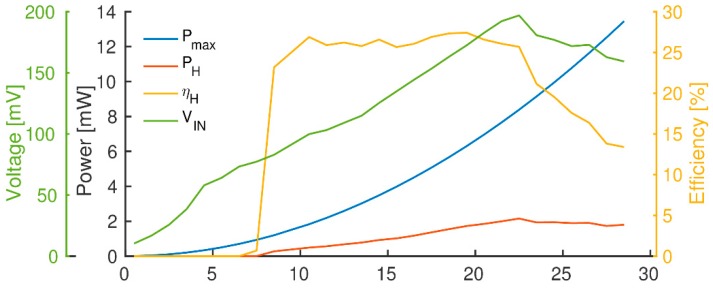
Evaluation of harvested TEG power for a different delta of temperatures (x-axis, Kelvin).

Figure 6 shows the experimental characterization of the TEG module and the used harvesting circuit for different temperature gradients in the thermal chamber. The x-axis shows the temperature gradient between the two TEG terminals measured using accurate (1% error) thermocouples. 

For temperature differences from 0 up to 28 °C, the graph shows the maximum power $P_{max}$ for matching load condition (2.78 Ω load) and the harvested power $P_{H}$. For $P_{H}$, all the conversion losses have already been considered, since it shows the power that is actually transferred to the storage capacitor. One can recognize the very low TEG harvesting efficiency $\eta_{H}$ of around 20%, even using state-of-the-art commercial harvesting chip. Moreover, notice that the harvesting circuit is not able to harvest energy if the TEG input voltage $V_{IN}$ is not at least 63 mV. To satisfy this condition, the TEG needs at least 5 °C temperature difference (only 20 μW), below this threshold the harvested power drops to zero. However, as shown in Table 5. it is possible to harvest between 20 μW (at 5 °C temperature difference) and 3 mW (at 28 °C temperature difference), which is a suitable value for our application.

**Figure 7:**
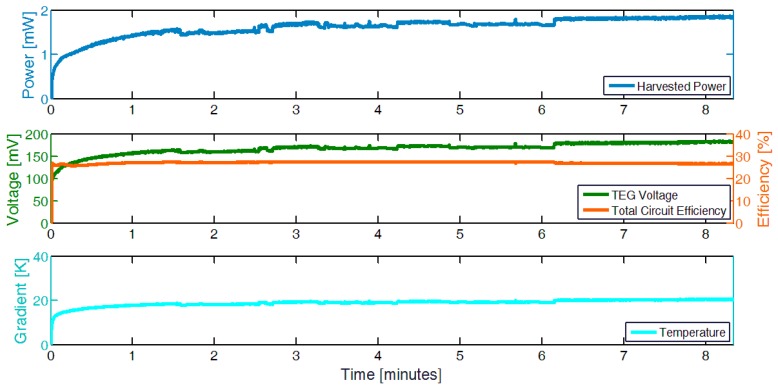
Estimated power harvested using the TEG module and the energy harvesting circuit, based on data collected during cutting tests where the TEG module was fixed on the motor.

To evaluate the effective power harvested, we measure the temperature gradient on the TEG during 8 minutes of cutting during the data acquisition tests on the Pedrazzoli SN270 motors. Using the accurately measured data of the TEG in the thermal chamber and the temperature gradient during the cutting process, we estimated the harvested power after the conversion circuit and the voltage of the TEG. Figure 7 shows the temperature gradient measured, the expected power harvested, and the TEG voltage in the application scenario. Moreover, it is possible to notice that the temperature increases during the process and becomes stable only after 6 minutes, giving more than 1.8 mW of power, although it is possible to start harvesting energy after only a few seconds. In fact, the experimental results show that after the initial 15 second period, the average effective energy harvested is higher than 1 mJ per second. 

**Figure 8:**
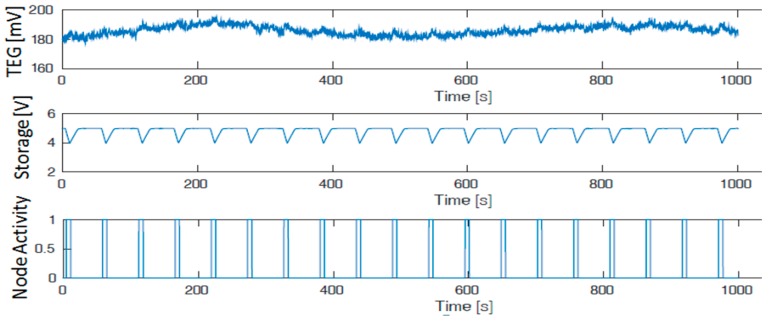
Perpetual work of the whole system supplied by the TEG energy harvester. TEG voltage, storage level, and wireless node activities. The picture shows only 15 minutes for legibility reasons but has been performed for several hours.

Finally, we evaluated the self-sustainability and the capability of the TEG module and the harvesting circuit to supply the whole system during data processing using a complete system testbed. The TEG module and the harvesting circuits have been deployed in a thermal chamber with the settings presented in the experimental setup section to achieve a 19 °C of the delta of the temperature on the two surfaces of the TEG. The TEG voltage, during the experiments, has been monitored as well as the storage capacitor voltage (4.7 mF). The node is configured to run the MAV 4 algorithm and send the data, executing it every ~55 seconds (~49 s sleep and ~6 s active). Figure 8 shows the storage level that is always to the maximum value and the capability of the system to be self-sustainable. The capacitor is fully recharged periodically, during the node’s sleep mode, and guarantees enough energy for the active mode. The testbed has been monitored for 5 hours keeping the same profile. For legibility reasons, we present only 15 minutes of data in Figure 8.

## 7. Simulations

In this section, we evaluate the capability of the system to be autonomous according to the model presented in Section 5 using MATLAB. We assume for simplicity the algorithm with four consecutive MAV evaluations, which is the most energy-hungry one. From Table 4, the total time needed to perform the classification and sending the data is less than 7 seconds, with a total energy of around 43 mJ. According to (5) to achieve energy neutrality the energy harvester has to be always greater or equal to the energy consumed, then the node can execute the algorithm only once every 43 s to have the time to acquire enough energy. In fact, assuming the node is out of the cold start, the energy acquired will be at least 1mJ per second, then, at least 43 s will be necessary to have 43 mJ of the necessary energy to perform one classification algorithm and one transmission. This is an appropriate value in such monitoring applications, where usually the duty cycle is around 0.01%, then the algorithms run only once per minute or even less. Moreover, in real applications, the data/information is sent only once per hour or day, and this increases the data processing sample rate further. To demonstrate our approach and the energy autonomy of the solution, simulations of (2), (3), (5) and (6) with the above presented experimental measurements were used. The simulation evaluates the storage level for 8 minutes according to the energy intake and the energy used in the data processing and radio communication.

**Figure 9:**
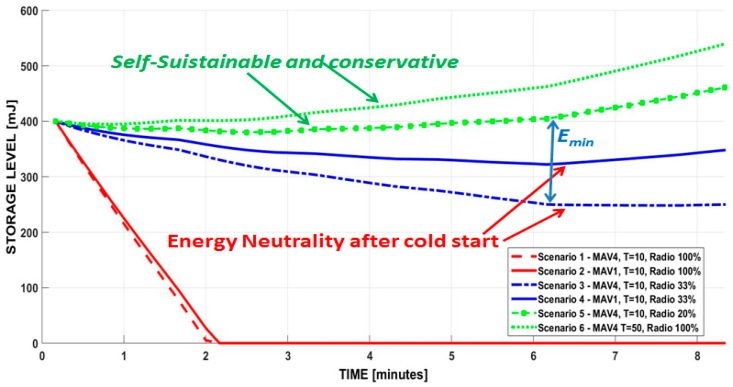
Simulations in the figure show the node’s storage level according to the intake energy and activities in six different scenarios.

As we are assuming to use an energy buffer, (6) was used to evaluate the level of available energy by the node at all times. We assume the initial energy level in a generic storage buffer is 400 mJ. Figure 9 illustrates the simulation results over six proposed scenarios where different duty cycles have been selected:

- *Scenario 1*: We assume T=10 s as the period, and the node executes the high accuracy classification algorithm with 4 MAV once per period, sending the data after every classification. Under these conditions, $T_{on}=7$ s and $T_{sleep}=3\;s$, as explained in Section 5, $T=T_{on}+T_{sleep}$.
- *Scenario 2*: We assume the same conditions of Scenario 1, but the classification algorithm is the single MAV evaluation which requires a smaller active period of the node $T_{on}=2\;s$, then $T_{sleep}=8\;s$. (T=10 s).
- *Scenario 3*: The same conditions of Scenario 1 (T=10 s, four MAV) are used, but the radio activity is reduced to save energy. In this scenario, the communication is active only once every 30 s (so $T_{tx\_scenario3}$= $T_{tx}/3$). This scenario allows to evaluate the influence of radio activity.
- *Scenario 4*: The single MAV algorithm evaluation with the same condition of Scenario 2 (T=10 s) but the activity of the radio is reduced to once every 30 s ($T_{tx\_scenario4}=T_{tx}/3$).
- *Scenario 5*: This scenario reduces the radio activity to be active only every 50 s ($T_{tx\_scenario5}=T_{tx}/5$), otherwise sharing the conditions of *Scenario 3.*
- *Scenario 6*: The algorithm is the four MAV and radio communication is again done after every classification, but with a period of T=50 s. As we still have $T_{on}=7\;$s for the selected algorithm, the system is in sleep mode for the remaining 43 s.

The red curves in figure are the scenario where the energy neutrality is not achieved (Scenario 1 and Scenario 2). The blue curves represent the energy neutrality (Scenario 3 and Scenario 4); finally, the green curves are the conservative scenario where energy is accumulated. When the radio activity is reduced the information is stored in the internal memory of the microcontroller and the data are sent in a single sending. 

The plots show that self-sustainability cannot be achieved either in Scenario 1 nor Scenario 2, since the storage level is zero after only around 2 minutes. This is because $\;T_{sleep}$ is too short to harvest enough energy to supply the node during the data processing phase. The plots demonstrate that it is possible to achieve an autonomous system by reducing the radio activity to 30% for both single classification (Scenario 4) and the high accuracy classification (Scenario 3) where more energy is required for each classification. The simulation shows that it is possible to make a conservative system where it is easy to store energy in the battery with the highly accurate algorithm when the radio activity is reduced to 20% (Scenario 5), i.e. sending data every 5 evaluations. In the conservative scenario the energy is accumulated in the buffer with a positive balance, then it is possible that the storage in a real application will be always fully charged. Finally, the simulation with Scenario 6 confirms that it is possible to achieve an autonomous system with accumulated energy, even sending a message after every classification, by increasing the period of T=50 s where the node is in the ultra-sleep mode for 43 s, saving energy to process and send the data. 

Simulation confirms that using storage is useful to guarantee the cold start of the system. In fact, as the TEG harvester starts to produce stable energy after a few minutes, the solution is energy autonomous only after that time. However, the wireless node can work using a part of the energy stored before and it is able to recharge the storage later when the energy harvester provides more energy. In these conditions, our system can work perpetually, and it can have a buffer of energy that is ready for the next cold start phase (assuming ultra-low leakage current of the storage). From these experimental results, it is also possible to evaluate the minimal energy (***E_min_)*** needed to guarantee the cold start condition to satisfy (7). The cold start is due to the fact that the energy acquired when the machinery is not active for long periods (i.e., one or more nights) is lower than after a few minutes of work activities. This value depends on the application scenario, as Figure 9 shows. For example, for Scenario 5 and 6, only 10 mJ and 20 mJ are needed to guarantee the cold start, while for scenario 3 at least 160 mJ are needed.

## 8. Conclusions

A novel accelerometer-based energy-neutral node for band saw blade wear detection is presented. The system uses ultra-low power sensors with wake-up radio capabilities that generate interrupts to wake-up the node on-demand which can significantly reduce activities on the main network, to achieve self-sustainability. Moreover, the node incorporates a standard Zigbee radio to send the information or when a critical situation is detected and needs to be transmitted to the remote host. The application leverages a low-complexity algorithm to perform high accuracy detection of blade wear. Due to the low cost and low power features of the system, it is suitable for industrial wireless sensor networks and the monitoring of industrial machinery.

By measuring the node’s power consumption, we confirmed the proposed solution achieves 100% algorithm accuracy at ultra-low energy consumption levels. Experimental results show the feasibility of autonomous energy system using only a small TEG generator, which was evaluated in the field during application tests. The node can be used in future applications to perform other vibration analysis tasks, by tuning the thresholds of the algorithm to “train” it to classify different kinds of vibration data.

## Figures and Tables

**Table 1:** Relationship between N and the size of the averaging window.

*N* (# Samples)	Time Interval Length for Single MAV [s]	Time Interval Length for 4 MAV [s]
2^9^	1.5	6
2^8^	0.75	3

**Table 2:** Classification accuracy.

Material and Blade Status	FFT	Time Domain with 4 MAV	Time Domain with 1 MAV
Brass, new blade	100	100	100
Brass, medium blade	100	100	**90**
Brass, old blade	100	100	100
Aluminum, new blade	100	100	100
Aluminum, medium blade	100	100	**87.5**
Aluminum, old blade	100	100	100
Nylon, new blade	100	100	**95**
Nylon, medium blade	100	100	**77.5**
Nylon, old blade	100	100	100
Steel rod, new blade	100	100	100
Steel rod, medium blade	100	100	**87.5**
Steel rod, old blade	100	100	100

**Table 3:** Threshold used in the on-board algorithm.

State of Blade	Threshold for MAV
New (N)	≤10
Medium (M)	>10 and ≤12
Old (O)	>12

**Table 4:** Node’s current and timing characteristics.

Device Mode	States	Radio	Current [mA]	Time [s]	Energy [mJ]
node sleep + radio wake-up	MSP430 (LPM3) and CC2530 (LPM3)	WUR	0.005	n/a	
data acquisition and processing	MCU on, accelerometer on, data sampling (ADC) on, and data processing	WUR	~0.155	~6 (4 MAV) ~1.5 (1 MAV)	2.79 (4 MAV) 0.7 (1 MAV)
data transmission	MCU on, radio TX, and data processing	CC2530 on	~34	0.382	39
data reception	MCU on, no processing, Radio RX	CC2530 on	~33	0.100	9.9

**Table 5:** Measured TEG power harvested for different deltas of temperature.

∆T	$P_{m}$ [μW]	Voltage [mV]	$P_{H}$ [μW]	TEG $E_{H}\;\mathrm{eff}.\;[\%]$
(0.0,1.0]	16.56	19.70	0	0
(1.0,2.0]	66.24	34.72	0	0
(2.0,3.0]	149.04	40.16	0	0
(3.0,4.0]	264.96	38.22	0	0
(4.0,5.0]	413.99	51.55	0	0
(5.0,6.0]	596.15	63.29	19.769	3.914
(6.0,7.0]	811.43	70.92	105.258	14.956
(7.0,8.0]	1059.83	78.07	202.844	21.680
(8.0,9.0]	1341.34	84.53	291.394	24.271
(9.0,10.0]	1655.98	90.94	395.881	26.416
(10.0,11.0]	2003.73	97.48	507.141	27.715
(11.0,12.0]	2384.61	104.64	623.331	28.408
(12.0,13.0]	2798.61	113.23	690.142	26.630
(13.0,14.0]	3245.72	119.40	727.095	24.059
(14.0,15.0]	3725.95	131.37	812.629	23.312
(15.0,16.0]	4239.31	139.37	1078.265	27.074
(16.0,17.0]	4785.78	147.48	1276.840	28.295
(17.0,18.0]	5365.37	155.39	1481.810	29.195
(18.0,19.0]	5978.09	162.04	1583.133	27.913
(19.0,20.0]	6623.92	169.28	1716.211	27.237
(20.0,21.0]	7302.87	179.92	1938.450	27.838
(21.0,22.0]	8014.94	188.41	2068.887	27.013
(22.0,23.0]	8760.13	197.34	2268.798	27.050
(23.0,24.0]	9538.44	207.75	2458.660	26.873
(24.0,25.0]	10349.87	211.12	2680.437	26.955
(25.0,26.0]	11194.42	205.83	2726.054	25.307
(26.0,27.0]	12072.09	198.83	2752.192	23.658
(27.0,28.0]	12982.88	196.21	3015.918	24.074
